# Automatic versus manual pressure support reduction in the weaning of post-operative patients: a randomised controlled trial

**DOI:** 10.1186/cc7695

**Published:** 2009-01-26

**Authors:** Corinne Taniguchi, Raquel C Eid, Cilene Saghabi, Rogério Souza, Eliezer Silva, Elias Knobel, Ângela T Paes, Carmen S Barbas

**Affiliations:** 1 Adult – ICU – Albert Einstein Hospital, Av. Albert Einstein 627-5 andar – São Paulo, SP, 05651-901, Brazil; 2 Pulmonary Division, University of São Paulo, Av Dr Eneas de Carvalho Aguiar 255-room 7079, São Paulo, SP, 05403-900, Brazil

## Abstract

**Introduction:**

Reduction of automatic pressure support based on a target respiratory frequency or mandatory rate ventilation (MRV) is available in the Taema-Horus ventilator for the weaning process in the intensive care unit (ICU) setting. We hypothesised that MRV is as effective as manual weaning in post-operative ICU patients.

**Methods:**

There were 106 patients selected in the post-operative period in a prospective, randomised, controlled protocol. When the patients arrived at the ICU after surgery, they were randomly assigned to either: traditional weaning, consisting of the manual reduction of pressure support every 30 minutes, keeping the respiratory rate/tidal volume (RR/TV) below 80 L until 5 to 7 cmH_2_O of pressure support ventilation (PSV); or automatic weaning, referring to MRV set with a respiratory frequency target of 15 breaths per minute (the ventilator automatically decreased the PSV level by 1 cmH_2_O every four respiratory cycles, if the patient's RR was less than 15 per minute). The primary endpoint of the study was the duration of the weaning process. Secondary endpoints were levels of pressure support, RR, TV (mL), RR/TV, positive end expiratory pressure levels, FiO_2 _and SpO_2 _required during the weaning process, the need for reintubation and the need for non-invasive ventilation in the 48 hours after extubation.

**Results:**

In the intention to treat analysis there were no statistically significant differences between the 53 patients selected for each group regarding gender (p = 0.541), age (p = 0.585) and type of surgery (p = 0.172). Nineteen patients presented complications during the trial (4 in the PSV manual group and 15 in the MRV automatic group, p < 0.05). Nine patients in the automatic group did not adapt to the MRV mode. The mean ± sd (standard deviation) duration of the weaning process was 221 ± 192 for the manual group, and 271 ± 369 minutes for the automatic group (p = 0.375). PSV levels were significantly higher in MRV compared with that of the PSV manual reduction (p < 0.05). Reintubation was not required in either group. Non-invasive ventilation was necessary for two patients, in the manual group after cardiac surgery (p = 0.51).

**Conclusions:**

The duration of the automatic reduction of pressure support was similar to the manual one in the post-operative period in the ICU, but presented more complications, especially no adaptation to the MRV algorithm.

**Trial Registration:**

Trial registration number: ISRCTN37456640

## Introduction

The weaning of mechanical ventilation or the removal of mechanical ventilation involves preparation of the patient and the progressive reduction of the ventilatory aid. As soon as the patients re-assume their capability to breathe on their own, the weaning process starts. The success of the weaning depends more on the patients' ventilatory capability than on the demand of the patient. In this manner, many factors need to be considered for weaning success: an adequate level of consciousness and respiratory drive; an adequate gas exchange with progressive decrement of the inspiratory and expiratory respiratory pressures; preserved respiratory muscle function and mechanics; and no severe metabolic or hydro-electrolytic disturbance [1].

The most popular weaning methods used are pressure support ventilation (PSV) and T-tube. Weaning through the T-tube is simpler but has some disadvantages such as the lack of an end expiratory pressure; the harsh change of the ventilation assistance; the lack of oxygen delivery control; and the lack of a proper ventilatory monitor [1]. One randomised controlled trial showed a superiority of the T-tube in relation to PSV and the necessity of a T-tube once a day to accelerate the weaning process [2,3]. Another controlled and randomised weaning trial has shown the superiority of PSV compared with T-tube and synchronised intermittent mandatory ventilation [4]. An attempt at PSV was made once a day and pressure support ventilation efficiently reduced the workload imposed on the respiratory muscles. The level of assistance can be gradually decreased until it only compensates for the additional work imposed by the endotracheal tube and the demand valve of the ventilator, at which time tracheal extubation can be performed. It can be used in association with positive end expiratory pressure (PEEP) and monitoring. Ely and colleagues [5] have shown that a daily attempt at spontaneous ventilation reduces the mechanical ventilation period. Smyrnios and colleagues [6] concluded that it was of great importance to identify and resolve the patients' weaning problems quickly, which is possible through systematic medical access to an organised multidisciplinary system of work. The prolongation of mechanical ventilation can lead to an increased risk of ventilation-associated pneumonia [7,8]; on the other hand, premature extubation followed by reintubation can increase the morbidity and mortality [9]. The major goal is to recognise readiness for extubation as soon and as reliably as possible [10]. Studies have shown that the duration of mechanical ventilation depends on a systematic approach in the weaning period for reducing the level of assistance and testing the possibility to resume spontaneous breathing [5,11,12].

The pressure support approach in mechanical ventilation weaning is designed to set the PSV mode to a level high enough to achieve a tidal volume (TV) of 8 mL/kg. This level of PSV is progressively reduced until a level of between five and seven is reached in accordance with the evaluation of the respiratory rate (RR), usage of accessory muscles and by the index RR/TV [13-18]. The reduction of the pressure support level can be made manually (by the physician or respiratory therapist) or automatically (by the ventilator itself according to an algorithm).

Automatic and computerised weaning using pressure support is based on an algorithm in the mandatory rate ventilation (MRV). This mode was first used in 1988 and consists of a ventilation mode that automatically decreases the PSV based on the RR target, during the weaning process. The RR target value represents the RR that the patient is expected to perform. In each cycle, the ventilator compares the RR target with the average RR, which was obtained from the last four cycles, with the limit ± three cycles per minute (CPM). If the average RR is higher than the RR target, the PSV will automatically be raised by 1 cmH_2_O. If the average RR is lower than the RR target, the PSV will automatically be decreased by 1 cmH_2_O. With an adequate PSV level the work of breathing decreases. If the PSV is overset, TV increases, work of breathing decreases and the RR will also decrease.

There is an inverse relationship between the PSV and the RR. Thus, this ventilation mode uses the RR as a parameter to adjust the PSV automatically. In this way, it is considered a safe ventilatory mode for stable patients [19,20]. When the PSV is adjusted correctly, the RR will be between 15 and 25 breaths per minute (bpm) physiologically, and the patient will breathe comfortably. Levels of PSV less than the patient requires will produce an increase in the work of breathing and an increase in RR. The opposite can also occur causing apnoea in the patient.

Chopin and Chambrin [21] alert us to the residual effect of the anaesthetics, which can reduce the RR, reducing the PSV precociously, and the minute ventilation which will lead to hypoventilation. However, there is a back up ventilation mode associated with MRV. The algorithm of MRV is based on the target RR. Our group wanted to test this algorithm in clinical practice to verify if the algorithm works with post-operative patients aiming to automatically wean these patients in our intensive care unit (ICU), thus facilitating the ICU routines.

We therefore hypothesised in this study that weaning with MRV is as fast and secure as the manual decrement of PSV during a weaning trial in the post-operative ICU mechanically ventilated patient.

The purpose of this study is to compare two methods of weaning from mechanical ventilation, using a prospective, randomised and controlled protocol. A comparison is made between weaning from PSV system by an automatic and computerised method (MRV) and the manual method of weaning, guided by the intensive care unit (ICU) staff, with regard to the time needed for the weaning process and the need of reintubation or non-invasive ventilation in post-operative patients.

## Materials and methods

We evaluated 158 postoperative patients admitted to the Albert Einstein Adult ICU in São Paulo, Brazil, who were over the age of 18 years, receiving mechanical ventilation after cardiac, thoracic, abdominal or orthopaedic surgery, from August 2002 to January 2004. We excluded patients who had neurological surgeries to avoid variations in the minute-ventilation in this patient population and patients with previous pulmonary disease or haemodynamic instability during the weaning of mechanical ventilation.

The study was approved by the ethical committee of our institution (CONEP-BRAZIL number 372/06) and was registered in ISRCTN-org as number 37456640. Signed informed consent was obtained from each patient or next of kin.

**Figure 1:**
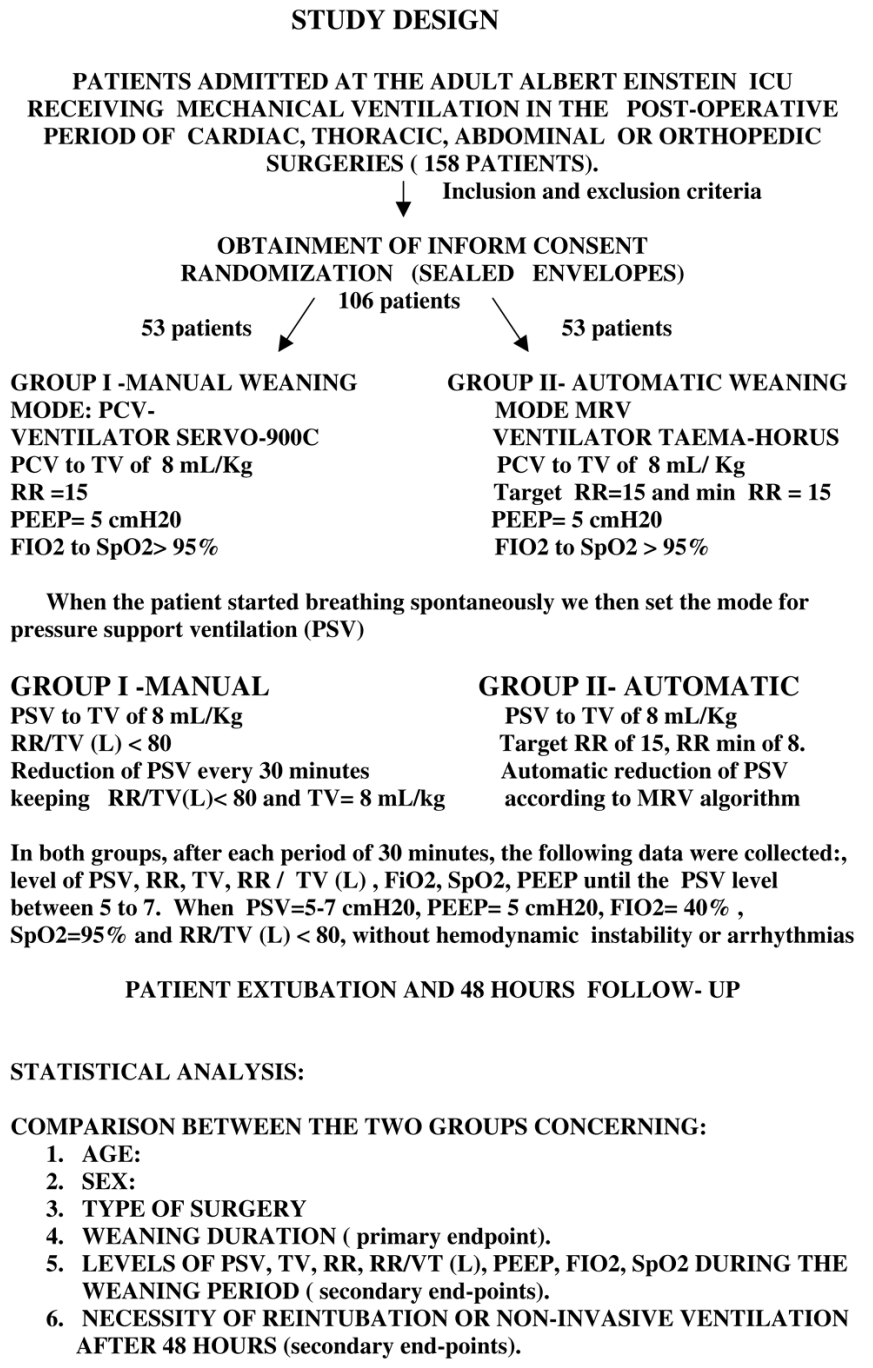
Study design. FiO_2 _= fraction of inspired oxygen; MRV = mandatory rate ventilation; PEEP = positive end-expiratory pressure; PCV = pressure controlled ventilation; PSV = pressure support ventilation; RR = respiratory rate; SpO_2 _= arterial oxygen saturation; TV = tidal volume.

When the patients arrived at the intensive care unit (ICU), they were still under the effect of the sedatives and under controlled ventilation. Therefore, to randomise patients, we randomly drew folded slips of paper from a large envelope. Each slip of paper provided an identification number and the assigned weaning method. Group I was manual weaning, guided by the ICU staff, ventilated with the Servo 900C^® ^ventilator (Maquet, Germany). Group II was MRV, automatic, computerised weaning, ventilated with the Taema-Horus Ventilator^® ^(Air Liquid, France) (Figure 1).

Group I was ventilated with a level of pressure controlled ventilation sufficient for a TV of 8 mL/kg; with a RR of 15 bpm; inspired oxygen fraction (FiO_2_) enough for oxygen saturation (SpO_2_) higher than 95%; PEEP of 5 cmH_2_O. Group II was ventilated using the same parameters of pressure-controlled ventilation, except that to obtain a respiratory rate of 15 bpm, in this mode, we set target RR as 15 bpm and the minimum respiratory rate as 15 bpm. This gave us the same parameters of ventilation in group I and II while the patient was recovering from the effects of sedation.

When the patient started breathing spontaneously we then set the mode for PSV.

Group I was ventilated with a level of PSV enough for a TV of 8 mL/kg; with the index RR/TV less than 80 L; FiO_2 _enough for SpO_2 _higher than 95%; PEEP of 5 cmH_2_O. The PSV was reduced every 30 minutes, by the ICU staff, keeping RR/TV less than 80 L and TV enough for 8 mL/kg. PSV could be reassessed and possibly decreased every 30 minutes aiming to make the manual weaning as close as possible to the automatic algorithm. Group II was ventilated with a level of PSV enough for TV of 8 mL/kg; with pressure support maximum (PS max) of 25 cmH_2_O; target RR of 15 bpm; minimum RR of 8 bpm; FiO_2 _enough for SpO_2 _higher than 95%; PEEP of 5 cmH_2_O. Setting the ventilator as above, in the MRV mode, the automatic reduction of PSV started.

In both groups, after each period of 30 minutes, the following data set was collected: level of PSV, RR, TV, RR/TV, FiO_2_, SpO_2 _and PEEP as far as the PSV level between 5 and 7. If the patient remained stable (without arrhythmias or haemodynamic instability), had adequate mental status and was capable of protecting the airway, with PSV between 5 and 7, PEEP of 5, FiO_2 _of 40%, SpO_2 _above 95%, RR/TV less than 80 L, they would be extubated. After the removal of the mechanical ventilation, the patients were observed for 48 hours to assess the need for reintubation or noninvasive ventilation.

The ICU co-interventions remained similar in both arms of the protocol during the study (sedation, analgesia, antibiotics, vasoactive drugs, if necessary) following our ICU standard of care routines.

### Endpoints

The primary endpoint was the duration of the weaning process, from the moment the patient started to breathe spontaneously until successful extubation. Secondary endpoints were: levels of pressure support, TV, RR, FiO_2_, SpO_2_, PEEP levels and RR/VT required during the weaning process, need of reintubation or need of non-invasive ventilation (Figure 1).

### Statistical analysis

For estimates of sample size calculation, a pilot study was performed with 40 patients that revealed a standard deviation (SD) of about 100 minutes in the two studied groups. Considering a study power of 80% and a p level (two-tails) of 0.05 it would be necessary to have 50 patients in each group to detect a mean difference of at least 60 minutes between the two groups.

Following the intention-to-treat principle, all randomised patients were included in these analyses. Continuous variables were summarised as means ± SD or median and interquartile ranges (IQR) when appropriate and categorical variables as absolute frequencies and percentages. The Chi-squared, Student's t and Wilcoxon tests were used to compare the two groups regarding categorical and continuous variables, respectively. In order to evaluate the respiratory parameters variations along time, a two-way repeated measure analysis of variance (ANOVA) was performed. The significance level was 0.05. The statistical package used for all analyses was the SPSS, version 11.0 (SPSS Inc, Chicago, IL, USA).

## Results

During the 17-month trial period, a total of 158 post-operative ventilated patients were screened, of whom a total of 106 patients were randomised to receive the intended treatments (53 in each study group). The characteristics of the patients are summarised in Table 1. Both the manual and automatic groups were similar in terms of age, gender and type of surgery. In total, 19 patients presented complications during the trial, four in the manual group (two because of haemodynamic instability; one because of apnoea and hypotension; one because of a refusal to participate in the study) and 15 in the automatic group (six patients because of haemodynamic instability; seven patients because the maximum PSV was reached; two patients because the PSV did not decrease). In the manual group the complications were equivalent to 7.5% of the group and in the automatic group they were equivalent to 28.3% of the group. According to the chi-squared test there was a significant difference between the two groups (p = 0.05). Therefore, 49 patients concluded the study in the manual group and 38 in the automatic group.

**Table 1:** Patients' characteristics

	**Total**	**Manual**	**Automatic**
**Age, years**	61 ± 13	61 ± 13	62 ± 14

**Sex, M/F**	69/37	36/17	33/20

**Type of surgery**			

**Cardiac**	41 (38.7%)	15 (28.3%)	26 (49.1%)
**Thoracic**	5 (4.7%)	3 (5.7%)	2 (3.8%)
**Abdominal**	52 (49.1%)	31 (58.5%)	23 (39.6%)
**Others**	8 (7.5%)	4 (7.5%)	4 (7.5%)

**TOTAL**	106 (100%)	53 (100%)	53 (100%)

Values of age are mean ± standard deviation. Values of gender are the number of patients of each group. In brackets are the percentages of patients in total and in each group.

**Figure 2:**
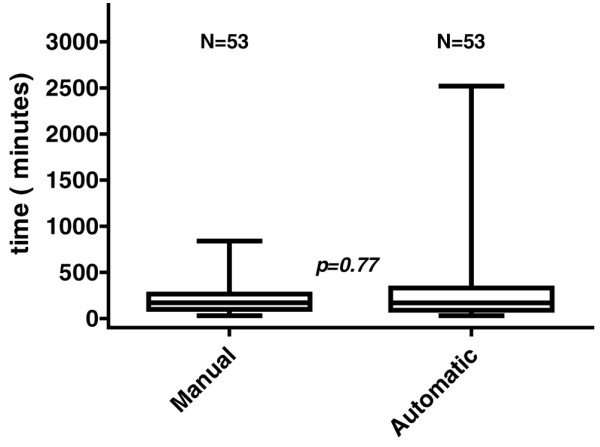
Boxplot of the median duration of weaning in manual and automatic weaning modes.

In the intention-to-treat analysis the mean duration of weaning until successful extubation was 221.04 minutes ± 192.07 minutes (minimum 30 minutes, maximum 840 minutes) in the manual group. The weaning duration of the automatic group was 271.32 minutes ± 369.38 minutes (minimum 30 minutes and maximum 2520 minutes). There was no statistic difference between the two groups (p = 0.375), the mean difference between the two groups was 50 minutes with the 95% confidence interval (CI) (-163 to 62) showing no superiority of automatic weaning. The median duration of weaning was 170 minutes (IQR 97.5 to 265 minutes) in the manual group and 168 minutes (IQR 90.5 to 330 minutes) in the automatic group (p = 0.777; Figure 2).

**Figure 3:**
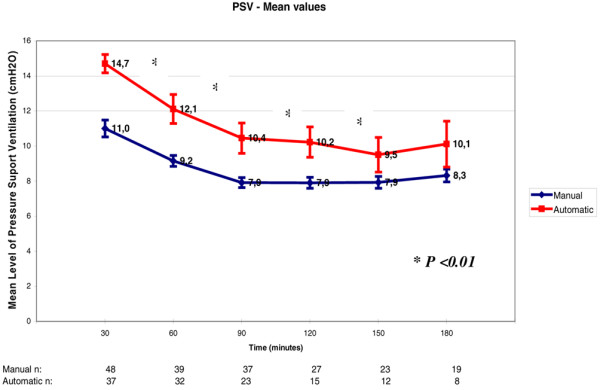
Pressure support ventilation (PSV) level variation throughout weaning. Each PSV refers to increments of 30 minutes in the weaning process. There was a significant difference between the automatic mode and the manual mode of weaning (p < 0. 01). The numbers at the bottom of the figure refer to the number of patients that stayed in the weaning trials through time.

Comparing decrement of pressure support in the automatic group to that of the manual mode we observed that the pressure support guided by the RR was significantly higher during the first three hours of the weaning period (p < 0.001 ANOVA; Figure 3).

**Figure 4:**
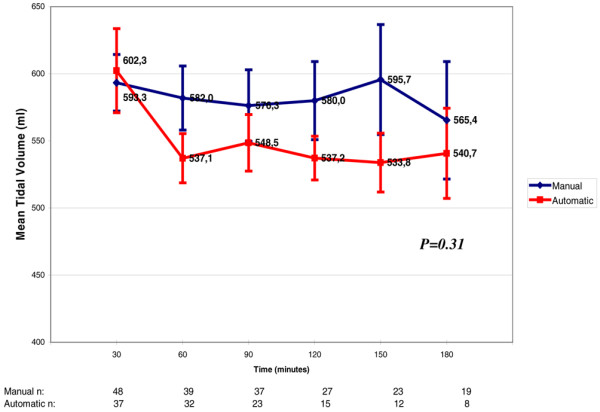
Variation of tidal volume throughout weaning. Each tidal volume value refers to increments of 30 minutes in the weaning process. There was no significant difference between the automatic mode and the manual mode (p = 0.31). The numbers at the bottom of the figure refer to the number of patients that stayed in the weaning trials through time.

The mean TV of both groups varied between 537 and 602 mL/kg. There was also no statistical difference between the two groups during the weaning period up to the point of successful extubation (p = 0.31; Figure 4).

**Figure 5:**
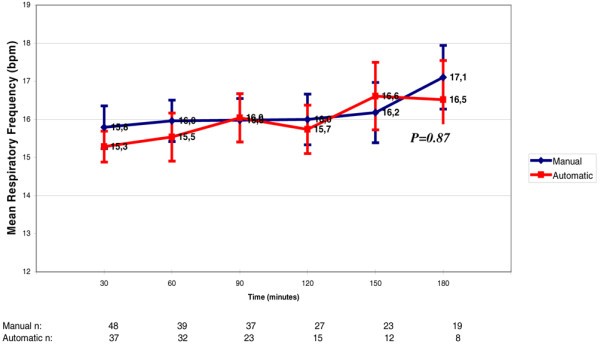
Variation of respiratory rate throughout weaning. Each respiratory rate value refers to increments of 30 minutes in the weaning process. There was no difference between the automatic mode and the manual mode (p = 0.86). The numbers at the bottom of the figure refer to the number of patients that stayed in the weaning trials through time. bpm = breaths per minute

The RR started at about 15 to 16 bpm in both groups. There was no difference between the two groups in the first 180 minutes of weaning (p = 0.87; Figure 5)

**Figure 6:**
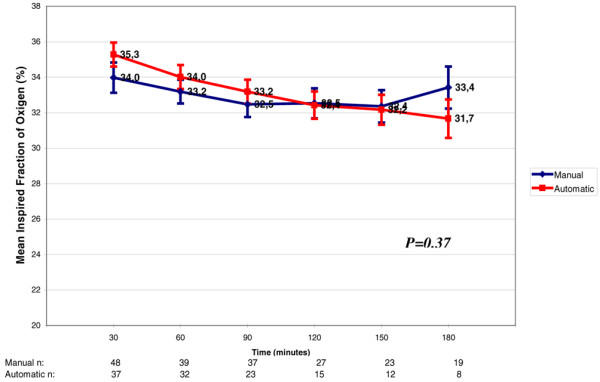
Variation in fraction of inspired oxygen (FiO_2_) throughout weaning. Each FiO_2 _value refers to increments of 30 minutes in the weaning process. There was no significant difference between the automatic mode and the manual mode (p = 0.37). The numbers at the bottom of the figure refer to the number of patients that stayed in the weaning trials through time. Decrement of FiO_2 _in both groups along time (p = 0.028)

The mean FiO_2 _during the weaning protocol was similar in both groups with a variation of 32% to 35% (p = 0.37). However, the effect of time was significant (p = 0.03) in both groups showing that the FiO_2 _decreased significantly in both groups during the first 180 minutes of the weaning process (Figure 6).

The SpO_2 _was maintained above 95% in both groups during the study period. (p = 0.16).

The PEEP value was maintained at about five to six in both groups throughout the weaning period (p = 0.06).

**Figure 7:**
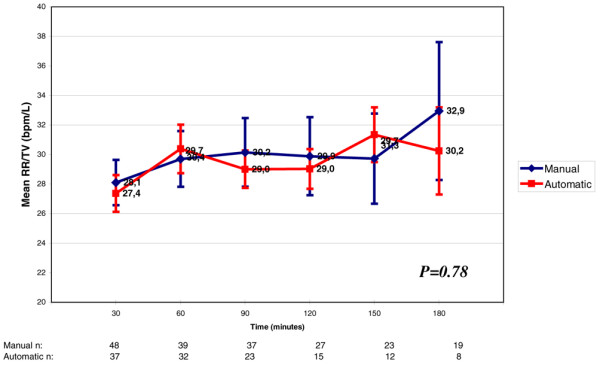
Variation of respiratory rate (RR)/tidal volume (TV) throughout weaning. Each RR/TV refers to increments of 30 minutes in the weaning process. There was no significant difference between the automatic mode and the manual mode (p = 0.78). The numbers at the bottom of the figure refer to the number of patients that stayed in the weaning trials through time.

The RR/TV index mean value varied from 27 to 31 L in the automatic group and from 28 to 33 L in the manual group during the study (p = 0.78; Figure 7).

No patient needed reintubation. Two patients needed non-invasive ventilation in the manual group after cardiac surgery (p = 0.51). We did not observe any serious adverse events in either of the groups.

### Complementary analysis

Nineteen patients presented complications during the weaning protocol. Four patients from the manual group and 15 patients from the automatic group (p < 0.05). Nine patients in the automatic group, or MRV, did not adapt to the set MRV algorithm. Trying to better understand why there was no adjustment to the MRV, we did a complementary analysis of those nine patients. The patients stayed in the MRV for 137 ± 135 minutes (95% CI = 5 to 450 minutes) before the staff decided to move to PSV. The weaning mean time of these nine patients was 329 ± 150 minutes (95% CI = 168 to 642 minutes), as shown in Table 2. This weaning duration process was greater than the weaning duration of the 39 patients that were well adapted to the MRV (157 ± 130 minutes; p = 0.0012).

**Table 2:** Details of nine patients excluded from the automatic group because of a lack of adaptation to mandatory rate ventilation

**Patients**	**Type of surgery**	**Time from the respiratory effort to change to PSV (minutes)**	**Weaning duration (minutes)**
**1**	Cardiac	60	228
**2**	Abdominal	90	258
**3**	Abdominal	60	288
**4**	Tracheoplasty	150	222
**5**	Cardiac	60	168
**6**	Burn debridement	6	330
**7**	Abdominal	450	642
**8**	Abdominal	240	330
**9**	Abdominal	120	498

	**Mean weaning duration**	**137.3**	**329.3**
	**Standard deviation**	**135.2**	**150.3**
	**Minimum to maximum**	**6 to 450**	**168 to 642**

PSV = pressure support ventilation

## Discussion

In this study, we evaluated the clinical suitability of a computer-driven automatic weaning system compared with the manual reduction of a PSV system in the postoperative period in an adult ICU. We concluded that automatic weaning is feasible providing the ICU staff (physicians and respiratory physiotherapists) spends this available time studying the weaning barriers of the ICU mechanically ventilated patients as a multidisciplinary team.

The computer-driven or automated weaning enables patients to interact with the ventilator and to adapt ventilation output in accordance with their individual and instantaneous needs [22]. In MRV, the PSV is set depending on the value of RR measured by the ventilator [23]. In our protocol, the duration of the weaning period up to successful extubation was similar to the manual group; however, the levels of pressure support were constantly higher in the automatic group than in the manual group. In order to keep the target RR at 15 bpm, the automatic algorithm chose levels of pressure support higher than those of the staff set by the RR/VT value (less than 80 L) in the manual group.

Lellouche and colleagues [10] in a multi-centre randomised trial showed that a computer-driven weaning protocol for patients admitted for more than 24 hours to the ICU, reduced mechanical ventilation duration and ICU length of stay, as compared with a physician-controlled weaning process. In contrast to this study, Rose and colleagues [24], in a recently published randomised controlled trial, found no benefit in using automatic weaning in a patient population made up predominantly of trauma and surgical patients. In our protocol we studied only post-operative patients, a population that has fewer respiratory diseases, comorbidities, and that can be more easily weaned and more successfully extubated from the mechanical ventilator.

One point to be considered is the number of patients that presented complications during the automatic weaning. Fifteen patients presented complications in the automatic mode compared with only four in the manual group, which is a significant difference (p = 0.05). In the manual group, the complications were haemodynamic instability (such as septic shock, haemorrhagic shock, cardiogenic shock or severe arrhythmias) in three patients, and one refusal to continue in the study. In the automatic group the complications were due to haemodynamic instability (five patients), one refusal to continue in the study and nine complications were related to the ventilator and the automatic mode of weaning. In six of the patients, the maximum pressure support of 25 cm H_2_O was reached and did not decrease, with the maintenance of a high level of pressure support. In three of the patients, even though the pressure support did not reach the maximum level, the patients were conscious and breathing comfortably but the pressure support did not decrease as expected to lower values of five to seven for the extubation.

The mean weaning time of these patients from the automatic group because of non adaptation to the mode, was 329.3 ± 150.3 minutes. In the automatic group, there was a significant difference in the mean weaning time between the group of patients who did not adapt to the MRV mode (329.3 ± 150.3) and the group of patients who did adapt to the MRV mode (157 ± 130; p = 0.0012). However, following the intention-to-treat principles, there was no difference between the automatic and manual groups considering the duration of weaning. From this, we can conclude that even though the automatic mode decreases the pressure support according to an internal algorithm, it should be watched by the ICU staff, as it may not reach its objective in all patients. One reason for this may be that the target RR was set too low for these patients. Maybe their comfortable RR was more than 15 bpm, in which case the PSV would not decrease, as it should do. Instead of the expected decrement of the PSV, to the contrary, it increases. Lellouche and colleagues [10] describe a 'comfort' zone which is defined primarily as a RR that can vary freely in the range of 15 to 30 bpm. He used this goal in a computer-driven protocol for weaning from mechanical ventilation with success. In another study, Dojat and colleagues [25] defined acceptable ventilation between 12 and 28 bpm for a clinical evaluation of a computer-controlled pressure support mode. Perhaps the target RR of 15 bpm was too strict, leading those patients to increase the PSV instead of decreasing it. For these patients the target RR should be higher than 15 bpm, the physiological RR chosen.

On the other hand, those patients who could not have the pressure support decreased, were patients who woke up agitated and confused. In these circumstances, they could not understand the staff requests or anything related to their respiratory care. In a pilot study of 20 children comparing clinician-driven and computer-driven system of weaning, Jouvet and colleagues [26] had three patients who could not be weaned from mechanical ventilation because the PSV value increased in the closed loop group. All of them had exacerbations of their lung disease; one also had to be transferred to normal ventilation due to agitation that caused a high variation in the RR and TV. According to their experience the closed loop weaning protocol is not adapted for patients with uncontrolled agitation episodes, especially patients with tracheobronchomalacia and infants under two years of age without sedation. It was also noted that this pilot study indicates that computerised decision-making seems reliable in a small population and requires validation in a larger sample of patients. For these patients, automatic weaning may not be the best solution.

The algorithms of pressure support reduction available in the micro processed ventilators in the ICU today have to be minutely understood. The choice of a respiratory rate of 15 bpm may have been below the ideal respiratory rate for an adequate functioning of the MRV algorithm for the total of our patients.

Other patients on automatic weaning had the value of pressure support decreased to the value of 5 to 7 cmH_2_O; however, there was a large fluctuation of pressure support during the weaning process. In some, instead of the pressure support decreasing gradually, it increased for a while and then decreased until the point of extubation. In the manual mode of weaning from the mechanical ventilation the pressure support was gradually decreased by the staff, the ventilatory aid was gradually diminished until extubation.

We can see from the cases above that when the patients were unable to conclude the study it was mostly due to shortcomings in the ventilators. It would be advisable to have more adjustments available on the ventilators to meet the patients' requirements. In the manual mode, the ICU staff adjusted the ventilation settings for their specific patient. Therefore, the manual mode allows individual settings that are not attached to a specific value such as the RR. The automatic mode does not allow settings in the same manner once the algorithm is responsible for the pressure support decrease.

Another point that should be noted is that during our study there were staff available to adjust the parameters every 30 minutes in the manual mode of weaning in order to optimise our manual weaning so that PSV could be reassessed and possibly decreased every 30 minutes as the automatic group decreased PSV every four cycles of ventilation. However, in reality, the ICU does not have this facility available in everyday practice. The other responsibilities of the staff do not allow a professional to be at the bedside adjusting the ventilator parameters every 30 minutes. Some studies have shown that clinical judgement is far from perfect and could tend to prolong mechanical ventilation [5,27]. We can say that in a way, the weaning time during our study was optimised compared with the usual practices of the ICU.

It should also be said that none of the patients in the study needed reintubation and only two patients in the manual group needed non-invasive ventilation after cardiac surgery after extubation. Both groups, after the exclusions discussed, had the same rate of successful weaning.

Wysocki and colleagues [28] in a review on closed-loop ventilation indicate that closed-loop ventilation is becoming stronger and that studies now available support the hypothesis that patient outcome is improving because of the use of closed-loop ventilation. In his opinion, in ICUs around the world driven by the triumvirate of cost-efficiency, quality and safety, closed-loop ventilation will become unavoidable. However, with regard to weaning, he also advises that more study on computerised-driven weaning is required before universal adoption.

### Study limitations

There were two important limitations in this study. There was only one Taema-Horus ventilator available for the study, making it impossible to randomise the patients when the Taema-Horus ventilator was already in use. This limited the number of patients that could be in the study. The other limitation was that in this study two types of ventilator were used. The manual weaning was performed on the Servo 900C^® ^ventilator and the automatic weaning was performed on the Taema-Horus ventilator.

## Conclusions

The duration of the automatic reduction of pressure support was similar to the manual one in the post-operative period in the ICU, but presented more complications, especially no adaptation to the MRV. The automatic reduction of pressure support can be used in clinical practice with the same efficacy as standard intensive care, in post-operative weaning from mechanical ventilation, if the patient is well adapted to the considered MRV algorithm.

## Key messages

• This randomised controlled clinical trial revealed that automatic reduction of pressure support available in mandatory rate ventilation is as effective as manual reduction of the pressure support in post-operative adult ICU patients, but presented more complications especially for patients with no adaptation to the MRV algorithm.

• The automatic reduction of pressure support is a feasible method of weaning and can be used in clinical practice if the patient is well adapted to the considered MRV algorithm.

• The use of automatic weaning could be an interesting and secure approach that could save ICU staff time and could improve the routine care of the post-operative adult ICU patients.

## Abbreviations

ANOVA: analysis of variance; BPM: breaths per minute; CI: confidence interval; CPM: cycles per minute; FiO_2_: fraction of inspired oxygen; ICU: intensive care unit; IQR: interquartile range; MRV: mandatory rate ventilation; PEEP: positive end-expiratory pressure; PS max: pressure support maximum; PSV: pressure support ventilation; RR: respiratory rate; SD: standard deviation; SpO_2_: arterial oxygen saturation; TV: tidal volume

## Competing interests

The authors have no competing interests to declare in relation to this manuscript.

## Authors' contributions

CT, RCE and CS made substantial contributions in the acquisition of the data. CT, RS, ES, EK and CSVB made substantial contributions to concept and design of the protocol. CT, ATP and CSVB were involved in the analysis and interpretation of the data and in drafting the manuscript.
